# Immune Response of Neonates Born to Mothers Infected With SARS-CoV-2

**DOI:** 10.1001/jamanetworkopen.2021.32563

**Published:** 2021-11-03

**Authors:** Maria Giulia Conti, Sara Terreri, Eva Piano Mortari, Christian Albano, Fabio Natale, Giovanni Boscarino, Giulia Zacco, Patrizia Palomba, Simona Cascioli, Francesco Corrente, Claudia Capponi, Mattia Mirabella, Ane Fernandez Salinas, Alessandra Marciano, Francesca De Luca, Ida Pangallo, Cecilia Quaranta, Claudia Alteri, Cristina Russo, Paola Galoppi, Roberto Brunelli, Carlo Federico Perno, Gianluca Terrin, Rita Carsetti

**Affiliations:** 1 Department of Maternal and Child Health, Policlinico Umberto I, Sapienza University of Rome, Rome, Italy; 2 Department of Molecular Medicine, Sapienza University of Rome, Rome, Italy; 3 Diagnostic Immunology Research Unit, Multimodal Medicine Research Area, Bambino Gesù Children's Hospital, IRCCS, Rome, Italy; 4 Diagnostic Immunology Unit, Department of Laboratories, Bambino Gesù Children's Hospital, IRCCS, Italy; 5 Research Laboratories, Bambino Gesù Children’s Hospital, IRCCS, Rome, Italy; 6 Department of Oncology and Hemato-Oncology, University of Milan, Milan, Italy; 7 Microbiology and Diagnostic Immunology Unit, Bambino Gesù Children's Hospital, IRCCS, Rome, Italy

## Abstract

**Importance:**

Although several studies have provided information on short-term clinical outcomes in children with perinatal exposure to SARS-CoV-2, data on the immune response in the first months of life among newborns exposed to the virus in utero are lacking.

**Objective:**

To characterize systemic and mucosal antibody production during the first 2 months of life among infants who were born to mothers infected with SARS-CoV-2.

**Design, Setting, and Participants:**

This prospective cohort study enrolled 28 pregnant women who tested positive for SARS-CoV-2 infection and who gave birth at Policlinico Umberto I in Rome, Italy, from November 2020 to May 2021, and their newborns. Maternal and neonatal systemic immune responses were investigated by detecting spike-specific antibodies in serum, and the mucosal immune response was assessed by measuring specific antibodies in maternal breastmilk and infant saliva 48 hours after delivery and 2 months later.

**Exposures:**

Maternal infection with SARS-CoV-2 in late pregnancy.

**Main Outcomes and Measures:**

The systemic immune response was evaluated by the detection of SARS-CoV-2 IgG and IgA antibodies and receptor binding domain–specific IgM antibodies in maternal and neonatal serum. The mucosal immune response was assessed by measuring spike-specific antibodies in breastmilk and in infant saliva, and the presence of antigen-antibody spike IgA immune complexes was investigated in breastmilk samples. All antibodies were detected using an enzyme-linked immunosorbent assay.

**Results:**

In total, 28 mother-infant dyads (mean [SD] maternal age, 31.8 [6.4] years; mean [SD] gestational age, 38.1 [2.3] weeks; 18 [60%] male infants) were enrolled at delivery, and 21 dyads completed the study at 2 months’ follow-up. Because maternal infection was recent in all cases, transplacental transfer of virus spike-specific IgG antibodies occurred in only 1 infant. One case of potential vertical transmission and 1 case of horizontal infection were observed. Virus spike protein–specific salivary IgA antibodies were significantly increased (*P* = .01) in infants fed breastmilk (0.99 arbitrary units [AU]; IQR, 0.39-1.68 AU) vs infants fed an exclusive formula diet (0.16 AU; IQR, 0.02-0.83 AU). Maternal milk contained IgA spike immune complexes at 48 hours (0.53 AU; IQR, 0.25-0.39 AU) and at 2 months (0.09 AU; IQR, 0.03-0.17 AU) and may have functioned as specific stimuli for the infant mucosal immune response.

**Conclusions and Relevance:**

In this cohort study, SARS-CoV-2 spike–specific IgA antibodies were detected in infant saliva, which may partly explain why newborns are resistant to SARS-CoV-2 infection. Mothers infected in the peripartum period appear to not only passively protect the newborn via breastmilk secretory IgA but also actively stimulate and train the neonatal immune system via breastmilk immune complexes.

## Key Points

**Question:**

What is the association of maternal SARS-CoV-2 infection with immune response in offspring in the first 2 months of life?

**Findings:**

In this cohort study of 21 mothers who tested positive for SARS-CoV-2 at delivery and their 22 newborns, there was 1 case of potential mother-infant vertical virus transmission and 1 case of horizontal virus transmission. Infants who received breastmilk during the first 2 months of life had significantly higher spike-specific salivary IgA antibody levels compared with formula-fed infants, and IgA spike immune complexes were detected in breastmilk.

**Meaning:**

Findings suggest that maternal protection goes beyond passive immunity, with immune complexes in breastmilk stimulating the active development of the neonatal immune system.

## Teaser

This cohort study characterizes the systemic and mucosal antibody production 48 hours and 2 months after parturition in mothers infected with SARS-CoV-2 and in their infants.

## Introduction

The World Health Organization has recently defined and categorized the timing of mother-to-child transmission of SARS-CoV-2.^1^ It may occur vertically (ie, in utero or intrapartum) or horizontally (ie, via droplets and respiratory secretions) in the early postnatal period.^2^ There is no evidence of SARS-CoV-2 transmission through breastfeeding.^3,4^ For this reason, breastfeeding is encouraged because it also provides nutritional benefits for the child and has a positive association with infant socioemotional development.^5^ Neonates born to women with asymptomatic or symptomatic COVID-19 are rarely infected by the virus either at birth or during the first month of life despite the close contact with their mothers with SARS-CoV-2–positive test results.^6^

The neonatal immune system is considered immature because the child’s adaptive immune response requires time to organize its architecture and generate protective immunity.^7^ During the first weeks after birth, the infant is therefore passively protected by maternal immunoglobulins transferred via the placenta during the last trimester of pregnancy.^8^ Passive protection continues after birth, with breastmilk containing maternal secretory immunoglobulin A (sIgA) antibodies mostly derived from the respiratory and intestinal maternal mucosal immune system.^9^

Specific SARS-CoV-2 maternal antibodies are transferred through the placenta during the last months of pregnancy and can be detected in the serum of the neonates born to naturally infected or vaccinated mothers.^10,11^ Similarly, maternal sIgA specific for SARS-CoV-2 is found in breastmilk of women who experienced COVID-19 or who received the vaccine.^12,13^ Thus, for COVID-19, as for many other infections, the mother uses her patrimony of antibodies to help the newborn transition from a state of maternal immunologic dependence to a state of immunologic self-sufficiency.

Whereas the development of specific antibodies and memory B and T cells requires months and even years to reach adult levels,^14^ immunity in the salivary glands starts early in life; IgA can be detected at birth and rapidly increases in the first 2 months of life.^15^ It remains unclear which mechanisms trigger the production of salivary IgA in fetal and neonatal life in the absence of infection.

In some cases, vertical transmission of antigen is possible, and it has been supposed that maternal cells or antigen-loaded microvesicles may traverse the placental barrier.^16^ Antigen transfer may also occur through breastmilk in antigen-antibody immune complexes. In a mouse model, IgG immune complexes induced an immune response in neonates.^17,18^

Salivary sIgA plays a fundamental role in the protection from respiratory viruses by blocking their attachment to epithelial cells.^19,20^ Adult patients with COVID-19 have high levels of specific and neutralizing sIgA in saliva.^21^ The SARS-CoV-2–specific salivary IgA has also been detected in children who were exposed and reacted to the virus but who never had a laboratory-confirmed positive test result for SARS-CoV-2 via nasopharyngeal swab (NPS).^22,23^

The neonatal immune response following in utero and early postnatal exposure to mothers with SARS-CoV-2–positive test results is still unclear. We hypothesized that the maternal immune response may not only passively protect the infant but also promote early-life–specific immune maturation. To investigate the immune mechanisms activated in neonates born to mothers with SARS-CoV-2 infection, we studied the systemic and mucosal antibody response of mothers and infants soon after delivery and 2 months later.

## Methods

This study followed the Strengthening the Reporting of Observational Studies in Epidemiology (STROBE) reporting guideline for cohort studies. The study was conducted in conformity with the World Medical Association Declaration of Helsinki^24^ for medical research involving human participants and was approved by the Ethical Committee of Policlinico Umberto I in Rome, Italy. Written informed consent from all parents was obtained at the time of enrollment. No one received compensation or was offered any incentive for participating in this study.

### Study Design and Population

This observational cohort study included mothers who tested positive for SARS-CoV-2 infection via NPS samples at delivery and their newborns. Included patients were consecutively admitted to Policlinico Umberto I, Sapienza University of Rome, Italy, and gave birth between November 2020 and May 2021.

### Treatment of the Mother-Infant Dyad

All mothers with SARS-CoV-2 infection were separated from their newborns immediately after birth and during their entire hospital stay owing to the shortage of health care workers and for space optimization.^25^ At hospital discharge, the dyad was reunited, and medical staff encouraged infant care and breastfeeding consistent with the Italian National Institute of Health COVID-19 safety guidelines, including handwashing and use of personal protective equipment when caring for the infant.

### Maternal and Neonatal SARS-CoV-2 Testing

Maternal SARS-CoV-2 infection was confirmed by the detection of the virus in NPS samples by using real-time reverse transcriptase–polymerase chain reaction performed at hospital admission.^26^ None of the mothers enrolled in the study was vaccinated for COVID-19 because vaccination of pregnant women was not allowed in Italy at the time of enrollment for the study. All neonates born to mothers with SARS-CoV-2–positive test results were also tested for SARS-CoV-2 using the same method as for the mother’s test immediately after birth and cleaning and again at 5 and 10 days of life.

### Collection of Biological Samples

Maternal and neonatal peripheral serum samples were collected 48 hours after delivery and 2 months later for the detection of anti–SARS-CoV-2-specific antibodies. Breastmilk samples were collected during the first 3 days after delivery from mothers with SARS-CoV-2–positive test results who were in stable clinical condition and were willing to pump milk. If the mother was breastfeeding 2 months after delivery, the breastmilk samples were also collected after nipple disinfection. For comparison, we measured specific antibodies in 7 control breastmilk samples obtained during the first week after parturition from mothers who delivered full-term healthy newborns and were never infected by SARS-CoV-2. All breastmilk samples were stored in sterile containers at −80 °C until further analysis.

Infant saliva samples were collected at 48 hours after delivery and 2 months later by aspiration with a sterile syringe (after removing the needle) from the oral cavity of all enrolled infants. Saliva samples were collected just before a meal (approximately 3-4 hours after the previous breastfeeding) to avoid contamination with maternal IgA in the breastmilk. All saliva samples were stored at −20 °C until the time of testing. For comparison, we also collected 6 saliva samples from healthy neonates breastfed by mothers never infected by SARS-CoV-2. Newborns were in healthy clinical condition, and the samples were obtained during the first week after birth.

### Clinical and Demographic Data Collection

We recorded clinical and demographic data for the mothers, including age, self-reported race and ethnicity, type of delivery, onset and clinical symptoms of SARS-CoV-2 infection, chest radiologic findings, and course of infection. At the 2-month follow-up visit, we recorded information on the use of personal protective equipment, hygienic measures during infant care, and SARS-CoV-2 infection of cohabitants.

Neonatal clinical baseline characteristics, including gestational age, sex, NPS test results for SARS-CoV-2, and length of hospital stay, were recorded. At 2 months of life, during the follow-up visit, we recorded the clinical condition of the newborns, and we collected data on feeding methods.

### Detection of Specific SARS-CoV-2 IgG, IgA, and IgM in Serum, Saliva, and Breastmilk Samples

Human IgG and IgA antibodies against SARS-CoV-2 were evaluated in serum, saliva, and breastmilk samples in a semiquantitative test by using an anti–SARS-CoV-2 ELISA kit (EUROIMMUN) according to the manufacturer’s instructions. Mothers’ serum and breastmilk samples were diluted 1:100, and infants’ saliva and serum samples were diluted 1:10. Results were evaluated as previously published.^27^ SARS-CoV-2 IgM antibodies to the receptor binding domain (anti-RBD) were detected using an in-house enzyme-linked immunosorbent assay (ELISA) as reported in our previous work.^27^

### Total IgG and IgA Detection in Saliva and Breastmilk Samples

Total IgG and IgA antibodies were detected in both neonatal saliva and breastmilk samples by ELISA. In brief, 96-well plates were coated for 1 hour at room temperature with purified goat anti-human IgG or IgA antibodies (Jackson ImmunoResearch Laboratories). After washing with phosphate-buffered saline (PBS; final strength, 1×) containing 0.05% Tween and blocking with PBS (1×) containing 1% bovine serum albumin, plates were incubated for 1 hour with diluted saliva (1:100) or breastmilk (1:100) samples. Secondary antibodies were peroxidase-conjugated F(ab)_2_ fragments of goat anti-human IgG or IgA antibodies (Jackson ImmunoResearch Laboratories). The assay was developed with o-phenylenediamine tablets (Sigma-Aldrich). Optical density was measured using a microtiter plate reader at a wavelength of 450 nm, and immunoglobulin concentrations were calculated by interpolation with a standard curve based on serial dilutions of monoclonal human IgG or IgA antibodies (Jackson ImmunoResearch Laboratories).

### SARS-CoV-2 Molecular Detection in Breastmilk Samples

The presence of SARS-CoV-2 RNA in breastmilk samples was defined by quantifying genomic RNA and subgenomic RNA using a QX200 Droplet Digital PCR System (ddPCR; Bio-Rad). Total RNA was extracted from breastmilk samples (400 μL) using a QIAamp viral RNA minikit (Qiagen) by following the manufacturer’s instructions. The SARS-CoV-2 genomic RNA was quantified as previously published.^28^ The SARS-CoV-2 subgenomic RNA was quantified using assays adapted for the ddPRC system and targeting the envelope transcripts, nucleocapsid transcripts, and spike transcripts.^29^ Molecular detection of SARS-CoV-2 in breastmilk samples from 7 mothers who tested negative for SARS-CoV-2 infection (controls) was also evaluated.

### Antigen Detection in Maternal Breastmilk Samples

Breastmilk samples were centrifuged (twice for 5 minutes each at 2000*g*), and approximately 200 μL of the resulting supernatant was used for detection and quantitation of SARS-CoV-2 nucleocapsid protein antigens via a chemiluminescent enzyme immunoassay (Lumipulse G SARS-CoV-2 Ag assay with the LUMIPULSE G System; Fujirebio). To avoid false-negative results, an internal control (10 μL) obtained from breastmilk samples of 7 control mothers who were breastfeeding was resuspended with supernatant (1 mL). Antigenic tests were also performed using these samples.

### Detection of IgG and IgA Immune Complexes in Breastmilk Samples

We assessed breastmilk samples for the presence of IgG and IgA immune complexes. In brief, 96-well plates were coated for 1 hour at room temperature with IgM antibody against the SARS-CoV-2 spike RBD (1 μg/mL; InvivoGen). After being washed with PBS (1×) containing 0.05% Tween and blocked with PBS (1×) containing 1% bovine serum albumin, the plates were incubated for 2 hours at 37 °C with 7 serial dilutions of breastmilk. The plates were then incubated for 1 hour with peroxidase-conjugated F(ab)_2_ fragments of goat anti-human IgA or IgG antibodies (Jackson ImmunoResearch Laboratories). We used o-phenylenediamine tablets for development. Optical density was measured on a microtiter plate reader at a wavelength of 450 nm.

To quantify the amount of IgA or IgG in the immune complexes, in each plate, we included a standard curve for total IgA or IgG. In brief, 1 column of the plate was coated with either goat anti-human IgA or IgG. Serial dilutions of monoclonal human IgA (10 μg/mL) or IgG (15 μg/mL) antibodies were used as standards and developed with F(ab)_2_ fragments of goat anti-human IgA or IgG antibodies (Jackson ImmunoResearch Laboratories) and o-phenylenediamine tablets. Immunoglobulin concentrations were calculated by interpolation of the standard curve (Jackson ImmunoResearch Laboratories).

### Statistical Analysis

We summarized continuous variables as median values and ranges (minimum to maximum values). Qualitative variables are expressed as numbers and percentages. We used the χ^2^ test or the Fisher exact test for categorical variables, and the *t* test, Mann-Whitney test, or Wilcoxon test for paired and unpaired variables. A 2-sided value of *P* < .05 was considered statistically significant. Statistical analyses were performed with GraphPad Prism, version 8.0 (GraphPad Software), and IBM SPSS Statistics software, version 25.0

## Results

We enrolled 28 women (mean [SD] age, 31.8 [6.4] years; 25 (89%) non-Hispanic White race and ethnicity) with SARS-CoV-2 infection, and their 30 infants (2 twin pregnancies; mean [SD] gestational age, 38.1 [2.3] weeks; 18 [60%] male and 12 [40%] female). A total of 21 mothers and 22 newborns completed the study, with 7 dyads lost to follow-up.

The main clinical characteristics of enrolled mothers and neonates are reported in the Table. At the time of delivery, all mothers had SARS-CoV-2 infection confirmed by a positive NPS test result. The majority of the mothers had mild symptoms; only 3 mothers were admitted to the COVID-19 unit for worsening clinical condition. We reported 1 case of possible vertical transmission to an infant defined by a positive PCR test result for SARS-CoV-2 on NPS samples performed immediately after birth.^1^ All other newborns repeatedly tested negative for SARS-CoV-2 infection during their hospital stay. At hospital discharge, when the dyads were reunited, 22 mothers (79%) still had a positive NPS test result for SARS-CoV-2 infection (Table).

**Table.** Maternal and Neonatal Demographic and Clinical Characteristics

Characteristic	No. (%) of patients
Mothers	28 (100)
Age, mean (SD), y	31.8 (6.4)
Maternal race and ethnicity	
Asian^a^	3 (11)
Non-Hispanic White	25 (89)
Type of delivery	
Cesarean	14 (50)
Vaginal	14 (50)
Length of SARS-CoV-2 infection before delivery, mean (SD),d^b^	4.5 (4.1)
Asymptomatic	6 (21)
Symptomatic	22 (79)
Anosmia	5 (18)
Ageusia	5 (18)
Myalgia	6 (21)
Fever	12 (43)
Pneumonia	9 (32)
Dyspnea	1 (4)
Cough	5 (189)
Diarrhea	1 (4)
Vomiting	1 (4)
Cold	2 (21)
Positive NPS at the time of reunification with infant	22 (79)
Use of PPE during infant care at home^c^	27 (96)
Always	17 (61)
Sometimes	10 (36)
At least 1 cohabitant with SARS-CoV-2 infection	22 (79)
Newborns	30 (100)
Gestational age, mean (SD), wk	38.1 (2.3)
Sex	
Male	18 (60)
Female	12 (40)
In utero/intrapartum transmission	1 (3)
Postnatal transmission (up to 2 mo of life)	1 (3)
NPS test for SARS-CoV-2 infection at 0 d of life	
Negative	29 (97)
Positive	1 (1)
NPS test for SARS-CoV-2 infection at 5 d of life	
Negative	27 (90)
Positive	1 (1)
Not available	2 (7)
NPS test for SARS-CoV-2 infection at 10 d of life	
Negative	17 (57)
Positive	1 (3)
Not available	12 (40)
Length of hospital stay, mean (SD), d	12.6 (10)
Feeding at 2 mo of life	
Exclusive formula diet	13 (43)
Maternal milk and formula diet	17 (57)

Abbreviations: NPS, nasopharyngeal swab; PPE, personal protective equipment: surgical mask or N95 mask or gloves.

^a^ The country of origin for all 3 women was China.

^b^ Positive nasopharyngeal swab test or onset of symptoms.

^c^ Starting from the reunification with the newborn.

### Association of Peripartum SARS-CoV-2 Infection With Transplacental Transfer of Maternal Spike-Specific Antibodies

**Figure 1.**
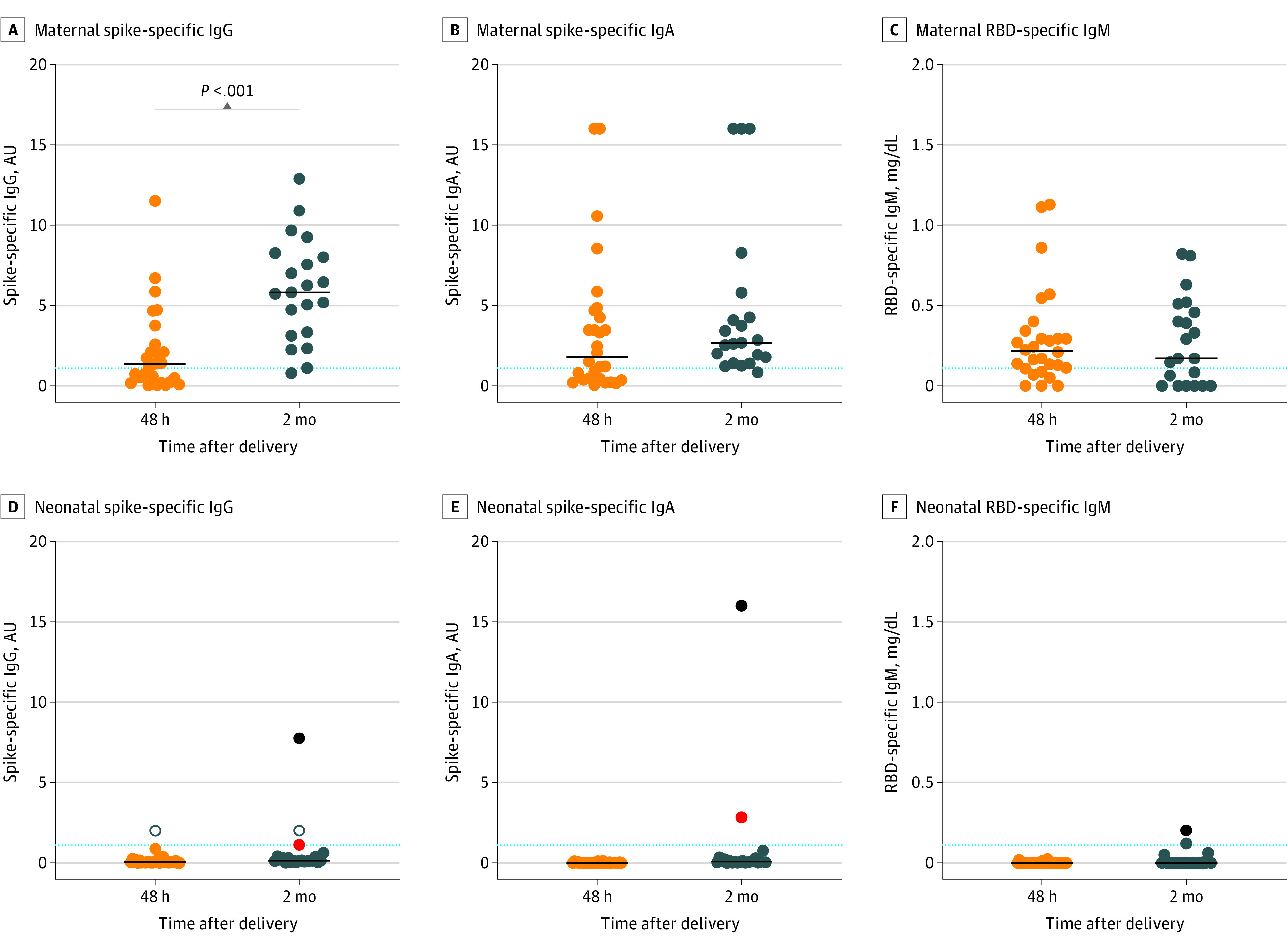
Maternal and Neonatal Serum SARS-CoV-2 Antibody Response A-C, Spike-specific IgG and IgA antibodies and receptor binding domain (RBD)–specific IgM antibodies detected in mothers 48 hours (n = 28) and 2 months (n = 21) after childbirth. D-F, Spike-specific IgG and IgA antibodies detected in infants and analyzed 48 hours (n = 30) and 2 months (n = 22) after birth. Open dots indicate the only case of a neonate in whom maternal IgG antibodies crossed the placenta; red dots, infant with possible vertical infection; black dots, infant with SARS-CoV-2 postnatal infection; dotted lines, detection threshold (1.1 arbitrary units [AU]). Median values are plotted, and statistical significance was determined using unpaired Mann-Whitney tests (compare ranks).

At 48 hours after delivery, antibodies specific for the virus spike protein were measured in the maternal serum. Spike-specific IgG antibodies were detectable in 16 of 28 mothers (57%), 17 (61%) of whom had spike-specific IgA, and 14 of whom had virus RBD-specific IgM (Figure 1A-C). At 2 months, the spike-specific IgA antibody level appeared higher (1.79 AU [IQR, 0.4-4.58 AU] vs 2.69 AU [IQR, 1.60-5.04 AU]; *P* = .11), reaching positivity in 19 of 20 mothers (95%), and the spike-specific IgG antibody level significantly increased (1.37 AU [IQR, 0.31-2.47 AU] vs 5.83 AU [IQR, 3.24-8.14 AU]; *P* < .001) in 19 of 20 mothers (95%), in agreement with the timing of the adaptive immune response. The RBD-specific IgM antibody level remained stable, without further increase (0.22 mg/dL [IQR 0.11-0.33 mg/dL] vs 0.17 mg/dL [IQR 0-0.48 mg/dL]; *P* = .80).

In the serum of newborns, SARS-CoV-2–specific IgG, IgA, and IgM antibodies were undetectable 48 hours after birth (Figure 1). We observed only 1 case in which maternal IgG antibodies crossed the placenta and were detected in neonatal serum. At 2 months of age, all infants were in good general clinical condition and showed no COVID-19–related symptoms since hospital discharge, as reported by their parents. Of 22 infants, 19 had no SARS-CoV-2–specific IgG, IgA, or IgM antibodies in their serum. The infant with a possible vertical infection had spike-specific serum IgA antibodies and barely detectable IgG antibodies. One infant had detectable serum SARS-CoV-2–specific antibodies of all isotypes, suggesting a possible asymptomatic postnatal infection.

### Spike-Specific IgA in Breastmilk of Mothers With SARS-CoV-2 Infection

**Figure 2.**
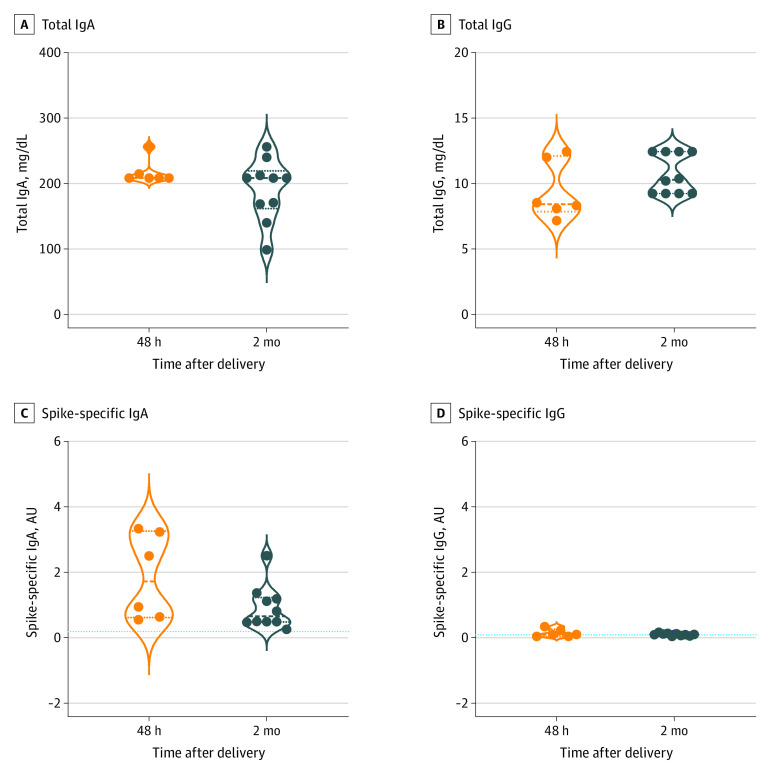
Breastmilk SARS-CoV-2 Antibody Response Violin plots showing total (A and B) and spike-specific (C and D) IgA and IgG antibody levels in maternal breastmilk 48 hours (n = 6) and 2 months (n = 10) after infant birth. Dotted lines indicate the mean value of spike-specific IgA (0.2 arbitrary units [AU]) and IgG (0.1 AU) antibodies detected in control breastmilk samples (n = 7). Median values are plotted, and statistical significance was determined using unpaired Mann-Whitney tests (compare ranks).

We collected 6 breastmilk samples 48 hours after delivery and 10 breastmilk samples at the 2-month follow-up visit because 11 of 21 mothers (52%) were not breastfeeding at that time. Total IgA and IgG antibody levels analyzed in breastmilk samples remained stable over time (Figure 2A, 2B). The IgA antibodies specific for SARS-CoV-2 spike protein were detectable in all breastmilk samples and appeared higher in 48-hour samples than in samples collected 2 months later (1.73 AU [IQR, 0.62-3.27 AU] vs 0.66 AU [IQR, 0.49-1.24 AU];* P* = .12) (Figure 2C). By contrast, spike-specific IgG levels remained persistently low. Spike-specific IgG and IgA antibodies and RBD-specific IgM antibodies were not detected in control breastmilk samples from 7 mothers who tested negative for SARS-CoV-2 infection.

### Infant Spike-Specific IgA in Saliva of Breastfed Infants

**Figure 3.**
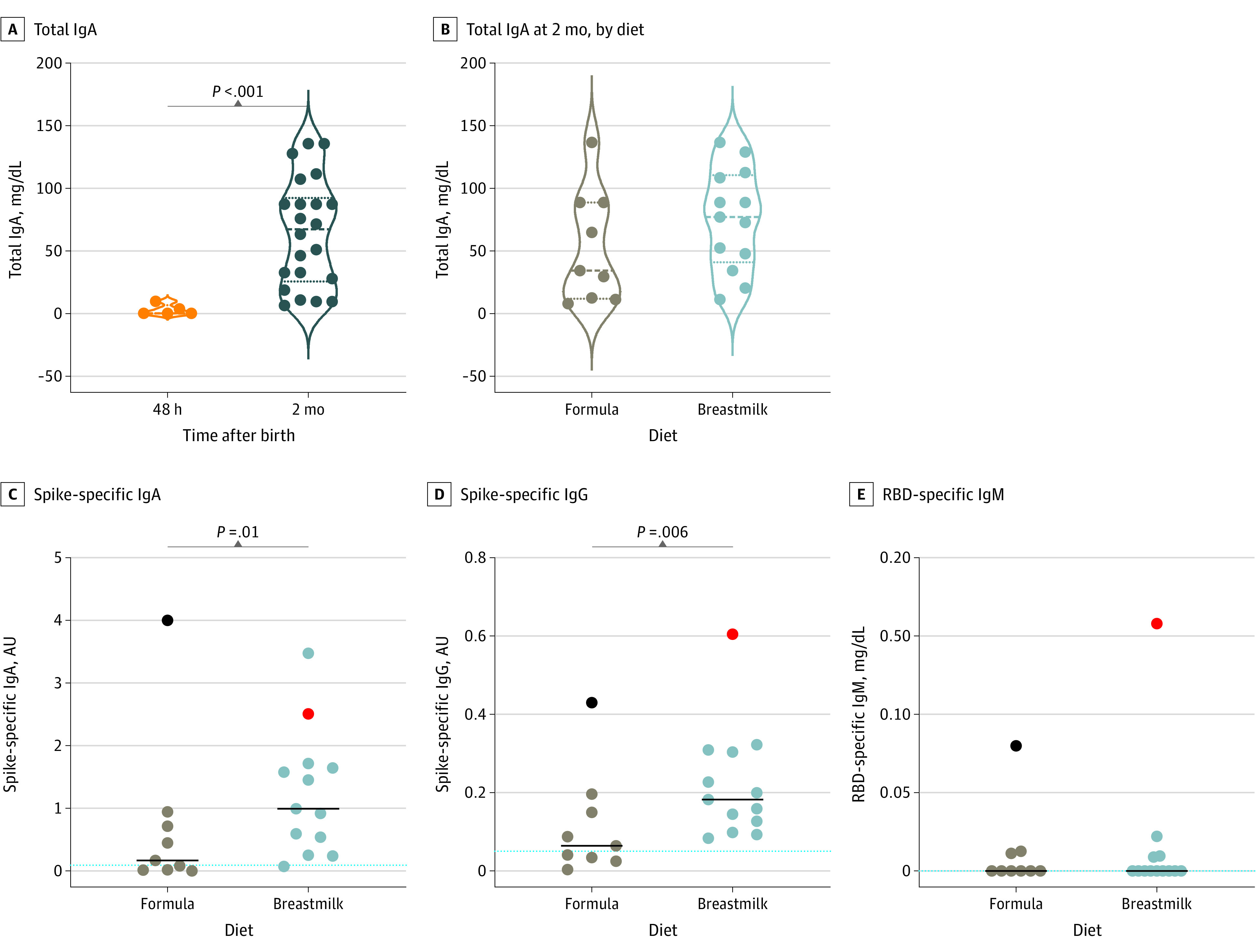
Antibody Detection in Saliva of Infants A, Violin plot showing total IgA antibodies in saliva of infants 48 hours (n = 5) and 2 months (n = 22) after birth. B, Total IgA antibody detection in neonatal saliva at 2 months of age, stratified according to diet. C-E, Spike-specific IgA and IgG antibody levels and RBD-specific IgM antibody levels in saliva according to diet (9 infants had an exclusive formula diet; 13 infants were breastfed). Red dots indicate infant with possible vertical infection; black dots, infant with a postnatal SAR-CoV-2 infection. Dotted lines indicate the mean values of spike-specific IgA (0.09 arbitrary units [AU]), spike-specific IgG (0.05 AU), and RBD-specific IgM (0 mg/dL) detected in saliva of control infants (n = 6). Median values are plotted, and statistical significance was determined using unpaired Mann-Whitney tests (compare ranks).

To investigate mucosal immunity in infants, we measured total IgA and spike-specific antibodies in saliva samples collected at 48 hours and 2 months of life. We found that total IgA levels significantly increased in all infants at 2 months of life (0 mg/dL [IQR, 0-6.64 mg/dL] vs 67.50 mg/dL [IQR, 25.80-92.40 mg/dL]; *P* < .001) (Figure 3A), consistent with the ontogeny of the mucosal immune system.^30^

At 2 months of age, the levels of total IgA antibodies in the saliva were comparable in infants who were breastfed and in infants who had received formula (Figure 3B), but breastfed infants had significantly higher levels of SARS-CoV-2 spike-specific IgA (0.99 AU [IQR, 0.39-1.68 AU] vs 0.16 AU [IQR, 0.02-0.83 AU];* P* = .01) and IgG in the saliva (0.17 AU [IQR, 0.11-0.28 AU] vs 0.05 AU [IQR, 0.03-0.13 AU; *P* = .006) (Figure 3C and D). However, RBD-specific IgM remained undetectable (Figure 3E). The infant with the potentially vertically transmitted infection and the infant with the postnatal infection had high levels of all isotypes of specific antibodies (Figure 3C-E). Because both infants were infected with SARS-CoV-2, they were considered outliers compared with other newborns and were thus not included in statistical analysis. Spike-specific IgG and IgA and RBD-specific IgM antibodies were also measured in saliva samples from 6 control infants whose mothers had NPS tests negative for SARS-CoV-2 infection, and these immunoglobulins were not detected. Viral transmission via breastmilk has not been detected in the recent studies, and those results are consistent with our findings showing that the virus was detected neither by digital PCR nor by the presence of the viral nucleocapsid by antigenic tests (eFigure and eTable in the Supplement).

### SARS-CoV-2 Spike-Specific IgA Immune Complexes in Breastmilk of Mothers

Antigen-antibody immune complexes are potent immune modulators. To explain the presence of spike-specific IgA antibodies in infant saliva, we investigated whether breastmilk samples contained immune complexes able to stimulate specific mucosal immunity in newborns.

**Figure 4.**
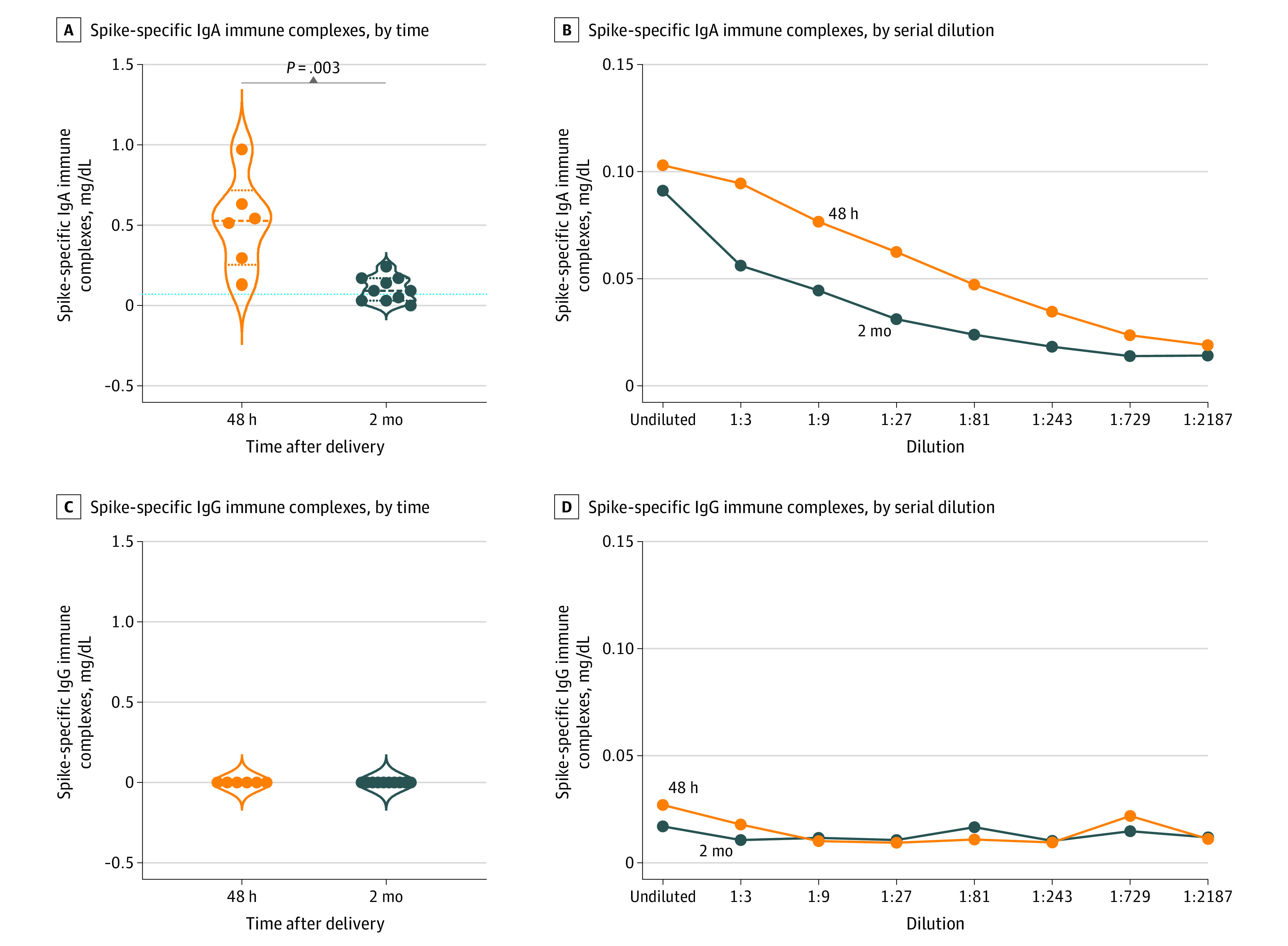
Virus Spike-Specific IgA and IgG Immune Complexes Detected in Breastmilk A and C, Levels of spike IgA and IgG immune complexes 48 hours (n = 6) and 2 months (n = 10) after delivery in the breastmilk of enrolled mothers with SARS-CoV-2–positive tests. Dotted lines indicate the mean values of spike-specific IgA (0.07 mg/dL) and IgG (0 mg/dL) immune complexes detected in breastmilk of 7 women with negative SARS-CoV test results. B and D, Serial 3-fold dilution curves, starting from undiluted samples, are shown. Median values are plotted, and statistical significance was determined using unpaired Mann-Whitney tests (compare ranks).

We found spike IgA immune complexes in all breastmilk samples analyzed. The levels of the immune complexes were significantly higher 48 hours after parturition (0.53 AU; IQR, 0.25-0.39 AU), when the infection was in the active phase, than they were 2 months later (0.09 AU; IQR, 0.03-0.17 AU), although IgA immune complexes remained detectable (*P* = .003) (Figure 4A). By contrast, IgG spike immune complexes were not detected in any breastmilk sample analyzed (Figure 4C). The IgG and IgA spike immune complexes were not detected in breastmilk samples from 7 mothers who tested negative for SARS-CoV-2 infection.

## Discussion

The results of this cohort study indicated that pregnant women who were infected with SARS-CoV-2 at the time of delivery lacked serum antibodies specific for the virus spike protein and were thus unable to transfer protection to the fetus. With 1 exception of vertical transmission, all offspring had neither a positive NPS test result nor COVID-19 symptoms, notwithstanding the lack of passively transferred maternal antibodies. The neonates were exposed again to the infection when they returned home with their closest contact, the mothers, most of whom tested positive for the virus at that time. None of the infants, with 1 exception, developed spike-specific antibodies in the serum at 2 months of age, indicating that they had never been infected with the virus (Figure 1D-F). Breastfeeding is encouraged after COVID-19 infection because breastmilk contains not only nutrients but also sIgA antibodies specific for the spike protein.

We also found that the saliva of the infants contained IgA specific for SARS-CoV-2 but only if they were breastfed, suggesting that maternal breastmilk stimulated the mucosal immune response in infants. We found that breastmilk did not contain the genetic material of the virus or the viral nucleoprotein. We hypothesized that small amounts of free spike protein in breastmilk were captured by maternal IgA and formed immune complexes able to stimulate the newborns’ immune response. To test this hypothesis, we developed an in-house ELISA and showed the presence of spike IgA immune complexes in maternal breastmilk. By comparison with a total IgA standard curve, we calculated that the IgA level in the immune complexes was 1000 times less than the total IgA level in the breastmilk. The immune complexes were more abundant in breastmilk samples collected 48 hours after parturition than in samples of breastmilk obtained at 2 months when the mother had recovered from the infection. The IgA antibodies detected in the saliva of the infants were more concentrated than in breastmilk. Salivary spike-specific IgA was also detected in 2 infants, 1 who was not breastfed for 1 week and 1 who was not breastfed for 1 month. Our results suggest that breastfeeding not only offers passive protection but also actively immunizes the child. Immune complexes have been proposed for the development of therapeutic and preventive vaccines, and they have been shown to stimulate B and T cellular responses.^31^

Because immunoglobulins present in saliva represent mucosal immunity, it is possible that the same response is diffused along the intestine of the infant.^30^ Our results indicated that immune complexes were formed in SARS-CoV-2 infection, but it is possible to imagine that the breastmilk may contain different types of immune complexes able to stimulate or tolerize the mucosal immune system of the infant. Further studies are necessary to confirm and expand our findings and to establish the extent of the phenomenon and the duration of immunity induced by breastmilk.

Our study showed that, in addition to the recognized role of breastmilk IgA antibodies in the control of infection and regulation of the microbiota community, immune complexes may also actively stimulate immunity in infants, preparing them for defense against the pathogens recently encountered by the mother. Thus, the mother instructs the immune system of the infant for protection and prevention, necessary when her transferred antibodies will decrease in the serum (±3 months of age)^32^ and breastfeeding will be interrupted.

Neither IgA antibodies present in the breastmilk nor the presence of IgA immune complexes explains the resistance of infants to SARS-CoV-2 infection because infants who were not breastfed did not develop the infection. Thus, other characteristics of the neonatal immune system play a role in this pandemic.^33^

Our study results cannot explain why neonates are protected from SARS-CoV-2 infection. However, by studying the mother-infant dyad in the unique “immunologic” condition of pregnancy, we offer a new strategy used by the mother’s immune system to help transition the newborn from a state of immunologic dependence to a state of immunologic self-sufficiency. Although the maternal immune system provides protection to the newborn through the transfer of maternal antibodies, there is also evidence that the fetus may already be exposed to antigenic stimuli during pregnancy^34^ and through exposure in utero to maternal antigens (active immune protection).^35^ Here, we showed that this process of priming the developing immune system continues after birth via breastmilk through the transmission of immunogenic immune complexes. It was previously known that breastfeeding protects infants against infections via the transfer of maternal sIgA, anti-inflammatory factors, and immunologically active cells. Our results now suggest that breastmilk may also contribute by triggering the function and development of the neonatal immune system by active immunization.

### Limitations

Our study evaluated the mucosal immune response of infants born to mothers who tested positive for SARS-CoV-2 infection and assessed the implication of breastfeeding from an immunological standpoint. However, our results should be interpreted with caution considering the limited number of cases included and enrolled from a single center at Policlinico Umberto I in Rome, Italy. However, this is the referral center for pregnant women who tested positive for SARS-CoV-2 infection and serves a large area of the Lazio region in Italy and reflected the situation of the territory during a peak period of the epidemic. Consecutive enrollment of all pregnant women positive for the virus limited selection bias. Six of the women enrolled in the study had a positive NPS test but did not show any symptoms associated with COVID-19; thus, it was difficult to trace the beginning of the infection. However, only 1 of the newborns had detectable IgG antibodies in the serum at 48 hours, suggesting that there was insufficient time for the mother to produce and transfer antibodies through the placenta. An additional limit of the study is the lack of a control group throughout; however, we preferred not to obtain blood samples from uninfected healthy mothers and their infants, thus exposing them to an unnecessary and painful procedure. Blood samples obtained from neonates born to mothers with COVID-19 infection is required to inform clinical treatment. We considered it important to have controls for the most original experiments in which sampling did not involve the use of invasive techniques (ie, neonatal saliva aspiration and breastmilk collection).

## Conclusions

This cohort study found that SARS-CoV-2 spike–specific IgA antibodies were detected in the saliva of infants who received breastmilk and who were exposed in utero and in the early neonatal period to the virus but were never infected. Our findings indicate that the mother’s immune system stimulates and trains the neonatal immune system for active protection via delivery of breastmilk immune complexes, extending previous work showing that mothers provide passive defense to their newborns by transplacental passage of maternal IgG antibodies and by sIgA antibodies via breastmilk. The SARS-CoV-2 pandemic has shown that, although this pathogen, which had never before been encountered by humans, frequently infects adult and elderly individuals who may develop severe and even lethal disease, children and infants rarely have symptomatic and acute COVID-19. The resistance of children to SARS-CoV-2 infection and disease is the subject of intense ongoing research.^33^

## Notes

Supplement. **eFigure.** SARS-CoV-2 Molecular Detection in Breastmilk **eTable.** Detection and Quantitative Measurement of SARS-CoV-2 Nucleocapsid Protein Antigen
