# Supplementary material for: Immune Response of Neonates Born to Mothers Infected With SARS-CoV-2
Source: JAMA Netw Open. 2021 Nov 3;4(11):e2132563. doi: 10.1001/jamanetworkopen.2021.32563 (PMC8567114; doi:10.1001/jamanetworkopen.2021.32563)
Supplement: Supplement. — eFigure. SARS-CoV-2 Molecular Detection in Breastmilk eTable. Detection and Quantitative Measurement of SARS-CoV-2 Nucleocapsid Protein Antigen [file jamanetwopen-e2132563-s001.pdf]

Supplementary Online Content

Conti MG, Terreri S, Piano Mortari E, et al. Immune response of neonates born to mothers infected with SARS-CoV-2. *JAMA Netw Open*. 2021;4(11):e2132563. doi:10.1001/jamanetworkopen.2021.32563

eFigure. SARS-CoV-2 Molecular Detection in Breastmilk

eTable. Detection and Quantitative Measurement of SARS-CoV-2 Nucleocapsid Protein Antigen

This supplementary material has been provided by the authors to give readers additional information about their work.

© 2021 Conti MG et al. *JAMA Network Open*.

eFigure. SARS-CoV-2 Molecular Detection in Breastmilk

A

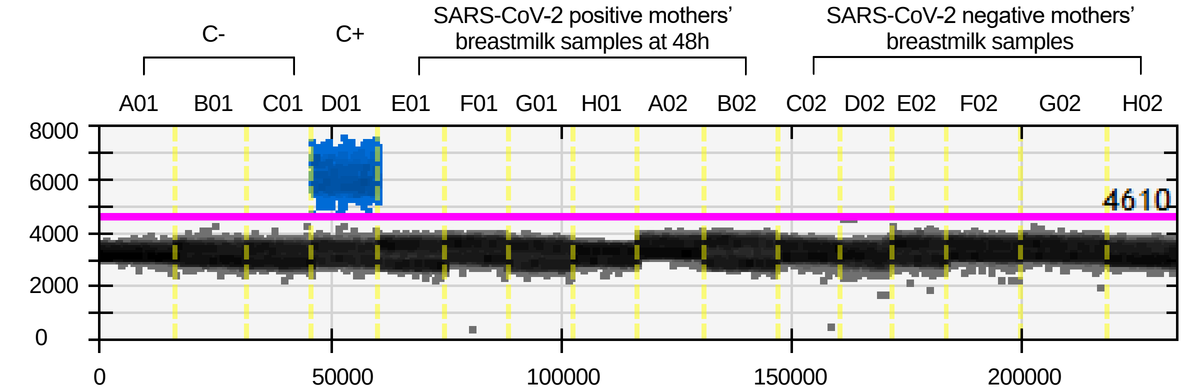

RdRp results

B

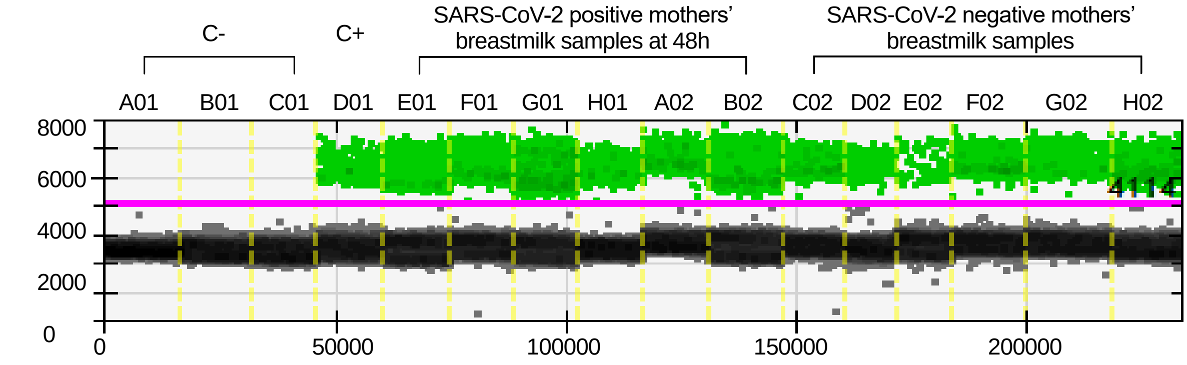

RNAseP results

(A) Amplification by digital droplet PCR of RdRp in the negative controls (C-), positive control (C+) and breastmilk samples. **(B)** Amplification by digital droplet PCR of RNAseP in the negative controls (C-), positive control (C+), and breastmilk samples. Results of SARS-CoV-2 subgenomic RNAs (sgRNAs) were superimposable and thus not shown.

© 2021 Conti MG et al. *JAMA Network Open*.

eTable. Detection and Quantitative Measurement of SARS-CoV-2 Nucleocapsid Protein Antigen

	Breastmilk samples	RLU	Ag Concentration (pg/mL)	Result
Ctrl	**1**	512	0.71	Ag not detected
	**2**	380	0.3	Ag not detected
	**3**	683	1.25	Gray zone
	**4**	277	0.01	Ag not detected
	**5**	246	0.01	Ag not detected
	**6**	337	0.16	Ag not detected
SARS-CoV-2 positive	**7**	282	0.01	Ag not detected
	**8**	903	1.24	Gray zone
	**9**	289	0.01	Ag not detected
	**10**	280	0.01	Ag not detected
	**11**	278	0.01	Ag not detected
	**12**	276	0.01	Ag not detected
	**13**	277	0.01	Ag not detected

**CTRL Neg**	271	0.01	NEG_Passed
**CTRL Pos**	51050	162.99	POS_Passed

Abbreviations: Ctrl, control (i.e., breastmilk samples of mothers never infected by SARS-CoV-2 collected during the first week after delivery); SARS-CoV-2 positive, breastmilk samples of mothers infected by SARS-CoV-2 collected 48h after delivery; RLU: relative light unit; Ag: nucleocapsid antigen; CTRL Neg: internal negative control; CTRL Pos: internal positive control.

© 2021 Conti MG et al. *JAMA Network Open*.
